# Relationship between the biomechanical properties of the cornea and anterior segment measurements

**DOI:** 10.6061/clinics/2018/e491

**Published:** 2018-09-14

**Authors:** Murilo Barreto Souza, Fabricio Witzel de Medeiros, Flavio Fernandes Villela, Milton Ruiz Alves

**Affiliations:** I Divisao de Oftalmologia, Hospital das Clinicas HCFMUSP, Faculdade de Medicina, Universidade de Sao Paulo, Sao Paulo, SP, BR

**Keywords:** Anterior Eye Segment, Corneal, Hysteresis, Scheumpflug, Bayes

## Abstract

**OBJECTIVE:**

To evaluate the relationship of biomechanical properties, corneal hysteresis and corneal resistance factor with age, sex and various corneal parameters measured with a Pentacam in normal subjects.

**METHODS:**

A total of 226 eyes from 113 patients were enrolled in this study. The subjects underwent Ocular Response Analyzer and Pentacam evaluations. A varying-intercept multilevel regression was implemented using Bayesian inference. The predictor variables were age, sex, central corneal thickness, corneal volume at a 7-mm diameter, anterior chamber angle and volume, anterior chamber depth, mean radius of the corneal curvature and corneal astigmatism.

**RESULTS:**

Corneal hysteresis ranged from 5.5 to 14.8舁mmHg (mean 10.42±1.74舁mmHg), and the corneal resistance factor ranged from 5.7 to 15.5舁mmHg (mean 10.23±1.88舁mmHg). No predictor variable other than gender and central corneal thickness had a significant correlation with either corneal hysteresis or corneal resistance factor. Corneal hysteresis was positively associated with female sex and with central corneal thickness, and corneal resistance factor was positively associated with central corneal thickness.

**CONCLUSION:**

Despite the associations found, only a small fraction of the variance in biomechanical measurements could be explained by the descriptors that were evaluated, indicating the influence of other corneal aspects on the biomechanical characteristics.

## INTRODUCTION

The spread of refractive surgery has increased interest in the relationships between biomechanical and structural characteristics of the cornea 1.

Modern imaging techniques have greatly improved corneal evaluation 2. The Pentacam (Oculus, Dutenhofen, Germany), an anterior segment tomographer, allows 3-dimensional study of the cornea and anterior segment to evaluate various anterior segment aspects, including corneal curvature and corneal thickness 1.

Previously, assessment of the biomechanical properties of the cornea was not easily achieved 3. The introduction of the Ocular Response Analyzer (ORA; Reichert Ophthalmic Instruments, Depew, NY, USA) allowed direct clinical evaluation of the biomechanical behavior of the cornea 4,5.

The aim of this study was to analyze the relationship of corneal resistance factor (CRF) and corneal hysteresis (CH) with age, sex and anterior segment parameters measured with the Pentacam in normal subjects.

## MATERIALS AND METHODS

A total of 226 eyes from 113 healthy subjects were enrolled in this study. Only patients with a best-corrected visual acuity (BCVA) of 0.0 LogMAR or better, with no use of a contact lens or no contact lens use for at least 72 hours, no ocular disease and evidence of a normal topographic pattern were included.

Each patient was subjected to a comprehensive ophthalmological examination, including refractive error measurement, BCVA assessment, and slit-lamp and fundoscopic examinations. Patients were also subjected, in the same visit, to anterior segment tomography (Pentacam) and corneal biomechanical evaluation with the ORA.

The FMUSP Institucional Review Board approved the study protocol.

### Measurements

Subjects underwent ORA and Pentacam evaluations by a trained technician, with the instruments calibrated by their respective developers.

The Pentacam is a non-contact optical system. It has a rotating Scheimpflug camera that takes up to 50 slit images of the anterior segment 6. The technique used in this study captured 25 slit images. Exams with excessive eye movement were discarded, and the examination was repeated.

During an ORA measurement, influenced by an air pulse, the cornea moves inward into a slight concavity and returns to its normal curvature, passing through two applanation periods. The two applanation pressures obtained differ due to corneal biomechanical properties. ORA also provides two intraocular pressure (IOP) measurements, a corneal compensated IOP (IOPcc) and a Goldmann-correlated IOP (IOPg). A waveform score is provided to reflect the quality of measurements. Only measurements associated with a waveform score greater than 5 were included 7.

### Data analysis

Bayesian inference describes the mathematical relationships between the “pre-trial” probability of an event and the “post-trial” probability, given the available data 8. Once a mathematical description for the data is defined, Bayesian inference can reallocate credibility across the variables whose values are sought, yielding probabilistic information for each possible result 9.

Analyzing data obtained from both eyes of the same subject without correction for correlation can underestimate standard errors and result in imprecise confidence intervals 10. To avoid this problem, one option is to reject data from one eye once analyses based on one eye per individual allow the use of standard statistical methods 10. This approach, however, leads to loss of valid data and reduces the potential power of the study 10,11. To avoid such loss of information and enable the inclusion of information from both eyes of the same subject, the statistical methods to be employed must account for the correlation between these paired observations 12. A random effects model accounted for this correlation 11 and was the statistical method used in this study.

A varying-intercept multilevel regression was implemented using Bayesian inference. The model contained a random intercept for participants and fixed effects for the predictor variables.

The response variables of interest were CH and CRF, and the predictor variables were age, sex, central corneal thickness (CCT), corneal volume at a 7-mm diameter, anterior chamber angle and volume, anterior chamber depth, mean radius of corneal curvature and corneal astigmatism.

In this study, the intercepts and slopes studied were assumed to follow a normal distribution. This normal distribution acted as a basis for the estimation of the intercept and slopes for each eye. To perform Bayesian inference, it is also necessary to specify the priors for the parameters of this normal distribution, µ and σ, known as hyperparameters. Additional knowledge can be used to build informative priors that capture previous subjective beliefs, or, when there are no strong beliefs, vague priors can be used 13. In this study, we evaluated models using vague priors. For the vague priors, we used a normal distribution (0,1000) for µ and a uniform distribution (0,100) for σ.

Estimates of the posterior distributions of the parameters of interest were obtained by Markov chain Monte Carlo (MCMC) procedures. The MCMC sampler was implemented in JAGS software 14. We used 500,000 iterations, discarding the initial 10,000 iterations to allow for burn-in. Trace plots and Gelman-Rubin convergence diagnostics were used to assess convergence of the generated samples. The posterior mean of the variance was used as the estimate of residual variance in the marginal *r*^2^ calculation. The marginal r^2^ describes the proportion of variance explained by the fixed effects.

The posterior distribution reflects the credibility of each possible parameter value. One way to summarize this information is to identify the values that are most credible, adding a total probability of 95%. This is called the highest density interval (HDI) 9.

For each explanatory variable evaluated, a 95% HDI, not including 0, indicated a meaningful predictor.

All statistical analyses were carried out with R (http://www.r-project.org) and JAGS 14.

## RESULTS

A total of 226 eyes from 113 patients were included in this study. The values of the ocular variables and demographics of the study population are presented in Table 1.

Tables 2 and 3 show the 95% HDI for each predictor variable in the CH and CRF models using vague priors. These data demonstrate that CCT exerts an influence on CRF and CH, as well as that CH is also influenced by sex. CH was positively associated with female sex and corneal central thickness, and CRF was positively associated with CCT.

Table 4 shows the variation of CH and CRF with IOP.

**Figure 1:**
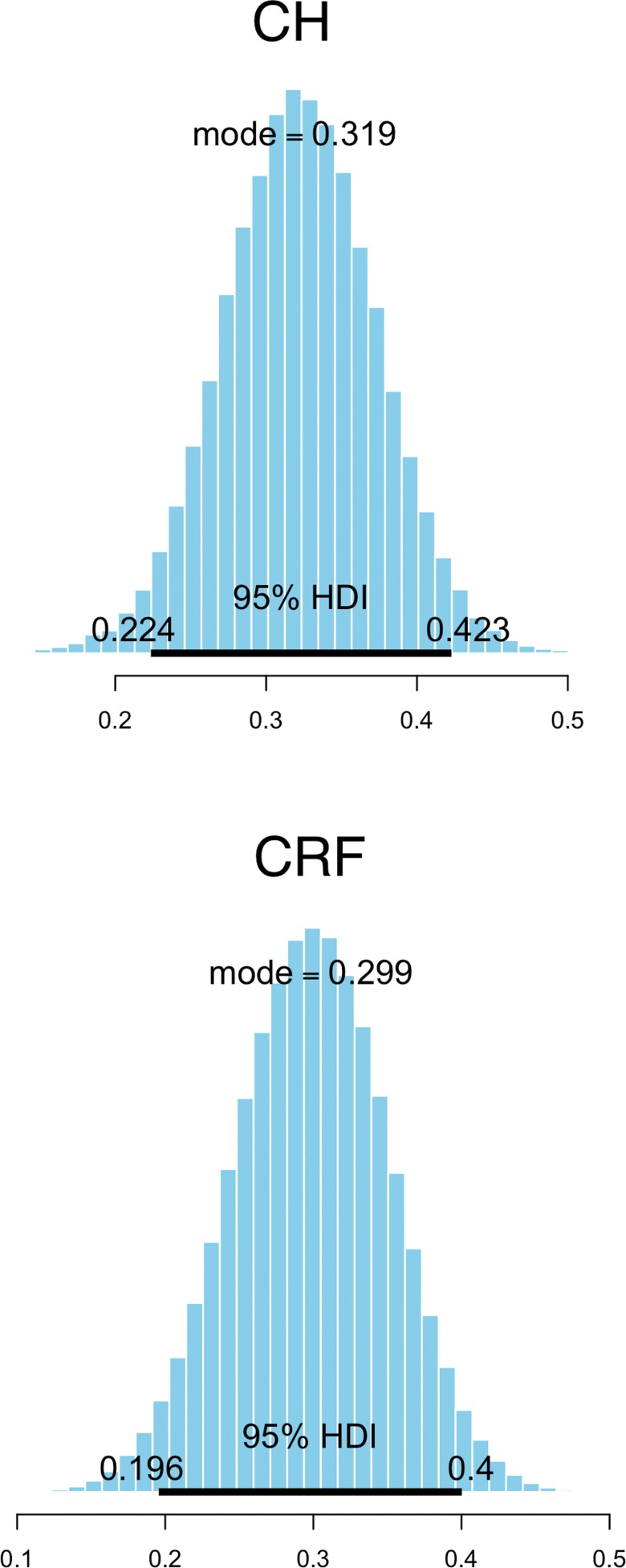
Posterior distribution of the estimated marginal *r*^2^ values. CH, corneal hysteresis; CRF, corneal resistance factor; HDI, highest density interval.

Figure 1 shows the posterior distribution of the estimated marginal *r*^2^ values.

## DISCUSSION

This study investigated the relationship of the biomechanical properties of the cornea with sex, age and anterior segment characteristics measured with the Pentacam using Bayesian analysis.

Although one of the major benefits of Bayesian inference is the ability to compare prior expectations with empirical data to determine whether they match, there are other advantages of Bayesian analysis. The inferences obtained from a Bayesian analysis reveal joint probabilities of combinations of parameter values, and there is no reliance on sampling distributions and p values to interpret the parameter estimates 9.

The relationships of anterior segment measurements with CH and CRF have been studied previously 4,5,15,16. There were previous reports on the influence of anterior chamber depth 17, astigmatism 18, corneal curvature and corneal volume 5 on CH or CRF. In this study, besides gender and CCT, no other predictor variable had a significant correlation with CH or CRF.

While CH reflects a direct measure of the corneal biomechanics, CRF was heavily weighted by elasticity once it was designed to have a maximum correlation with corneal thickness. Accordingly, the positive relationship between CCT and CRF observed in this study was highly expected and has already been described 3,19.

In accordance with our results, the CCT was found to be positively correlated with CH in many studies 19-22. The finding that CH reflects the viscous properties of the cornea could indicate that the viscosity increases as the corneal thickness increases. However, it is important to highlight that CH has also been shown to be reduced in patients with Fuch's endothelial dystrophy 23, a condition that causes a progressive increase in CCT. This fact could indicate the influence of other corneal characteristics on corneal biomechanical properties, such as the composition of the extracellular matrix or water content.

The relationship between IOP and corneal biomechanical properties is not completely understood 24.

The ORA measurements supposedly allow differentiation between CH and IOP 25. According to this theory, the hysteresis parameter should remain constant for the same set of corneas over a wide range of pressures 25. However, during ORA measurements, the IOP may represent an additional force that resists deformation and restores the cornea to its original position 26.

Sun et al. showed that in eyes with chronic primary angle-closure glaucoma, the CH was significantly lower than that in control eyes and that the CH level partially recovered after lowering of the IOP 27. The authors observed that although CH could be independent of IOP under normal physiological conditions, above 21 mmHg, higher IOPs could be associated with alterations in CH 27. A theory postulated to explain the IOP influence on CH suggests that at high IOP levels, corneal collagen fibers may already be significantly stretched, thus decreasing the difference between the ORA parameters P1 and P2 28.

A negative correlation between IOPcc and CH and a positive correlation between IOPg and CRF were observed in this study. Similar results have already been described 17. The IOP effect was not included in our multiple regression models, which may have influenced our results. A future study should further investigate the influence of IOP using different techniques for IOP measurement.

Estrogen influences corneal thickness and the biomechanical properties of the cornea 29. Changes in corneal curvature in women taking contraceptives or during pregnancy demonstrate this effect 30. Spoerl et al. 29 found a reduction in the corneal stiffness by 36% due to estradiol treatment. Although this reduction does not play an important role under normal conditions, it can be important in a biomechanically weakened cornea, such as corneas submitted to laser-assisted in situ keratomileusis procedures. In this study, female sex was positively associated with CH, a relationship described in previous studies 31.

There is indirect evidence to suggest that corneal biomechanics properties vary with age 32. The inverse relationship between age and incidence of keratoconus represents one example of this evidence 31.

Aging, probably influenced by a combination of oxidative stress and the formation of advanced glycosylation end products, results in cross-linking of proteins and reduced tissue elasticity 32. In the cornea, a reduction in mechanical compliance with age has already been described. Knox Cartwright et al. 34 showed that human corneal stiffness approximately doubles between the ages of 20 and 100. Pallikaris et al. 35 also found a positive correlation between ocular rigidity and age.

Our study did not reveal a correlation between age and CH or CRF, which could be due to the ethnic characteristics of the population or the small number of older subjects included in this study. Similar results were observed in other studies involving predominately young patients 5,15.

As indicated by the marginal r^2^ values, our results showed that only 20-40% of the variance regarding CH and CRF could be explained by variations in the evaluated explanatory variables studied. These results indicate the influence of other aspects on the biomechanical characteristics.

## AUTHOR CONTRIBUTIONS

Souza MB and Alves MR were involved in data collection, interpretation of the results and manuscript drafting. Villela FF and Medeiros FW reviewed the manuscript and contributed to interpretation of the results.

## Figures and Tables

**Table 1:** Values of the ocular variables and demographics of the studypopulation.

Variable	Mean	Std. Dev.	Range
Age	39	±17.6	11 - 77
Gender			
Female	68 (60%)		
Male	45 (40%)		
Corneal resistant factor, mmHg	10.23	±1.88	5.7 - 15.5
Corneal hysteresis, mmHg	10.42	±1.74	5.5 - 14.8
Corneal astigmatism, D	1.2	±0.94	0.2 - 4.8
Central corneal thickness, µm	547.02	±35.1	438 - 630
Corneal volume, mm^3^	26.64	±1.47	20 - 28.4
Mean radius of corneal curvature, mm	7.83	±0.25	7.22 - 8.47
Anterior chamber volume, mm^3^	177.57	±48.4	80 - 310
Anterior chamber depth, mm^3^	3.02	±0.46	1.95 - 4.76
Anterior chamber angle, ^o^	37.58	±7.5	18.8 - 65.4
Corneal compensated IOP, mmHg	15.38	3.5	6.9 - 25.6
Goldmann correlated IOP, mmHg	15.38	3.4	7.6 - 29

Std. Dev., Standard deviation; IOP: intraocular pressure.

**Table 2:** 95% highest density interval for each predictor variable in the CRF models using vague priors.

	95% HDI
Gender	-0.178 - 0.875
Corneal stigmatism	-0.379 - 0.036
Central corneal thickness	0.016 - 0.030*
Age	-0.022 - 0.009
Anterior chamber angle	-0.003 - 0.011
Anterior chamber volume	-0.004 - 0.002
Anterior chamber depth	-0.367 - 0.244
Mean radius of corneal curvature	-0.690 - 0.331
Corneal volume	-0.145 - 0.248

CRF, corneal resistance factor.

* meaningful parameter.

**Table 3:** 95% highest density interval for each predictor variable in the CH models using vague priors.

	95% HDI
Gender	0.054 - 0.995*
Corneal astigmatism	-0.246 - 0.172
Central corneal thickness	0.013 - 0.028*
Age	-0.025 - 0.002
Anterior chamber angle	-0.003 - 0.013
Anterior chamber volume	-0.005 - 0.002
Anterior chamber depth	-0.273 - 0.336
Mean radius of corneal curvature	-0.829 - 0.083
Corneal volume	-0.072 - 0.293

CH, corneal hysteresis.

* Meaningful parameter.

**Table 4:** Mean (standard deviation) corneal hysteresis and corneal resistance factors according to IOP group.

		IOPcc			IOPg	
		*CH*	*CRF*		*CH*	*CRF*
PIO	No.	Mean ±SD	Mean ±SD	No.	Mean ±SD	Mean ±SD
**< 10**	10	12.1±1.2	10.2±1.4	15	10.2±1.5	8.3±1.4
**10 - 15**	106	10.9±1.4	10.1±1.7	116	10.2±1.6	9.4±1.5
**15 - 20**	84	9.9±1.6	10.1±2	74	10.9±1.6	11.3±1.4
**>20**	26	9.0±1.8	10.8±1.8	21	9.7±2.2	11.8±1.9
P value for trend		<0.001*	0.204		0.337	<0.001*

SD, standard deviation; CH, corneal hysteresis; CRF, corneal resistance factor; IOPcc, corneal compensated intraocular pressure; IOPg, Goldmann-correlated intraocular pressure.

* Test for differences in trend between IOP groups.
